# Use of a Micronutrient Cocktail to Improve Metabolic Dysfunction-Associated Steatotic Liver Disease (MASLD) in Adults with Obesity: A Randomized, Double-Blinded Pilot Clinical Trial

**DOI:** 10.3390/medicina60081366

**Published:** 2024-08-21

**Authors:** Iulia Teodora Perva, Iulia Elena Simina, Renata Bende, Alexandru Cătălin Motofelea, Adela Chirita Emandi, Nicoleta Andreescu, Alexandra Sima, Adrian Vlad, Ioan Sporea, Cristian Zimbru, Paul Calin Tutac, Maria Puiu, Mihai Dinu Niculescu

**Affiliations:** 1 Department of Microscopic Morphology, Genetics Discipline, Center of Genomic Medicine, “Victor Babeș” University of Medicine and Pharmacy, Eftimie Murgu Sq., 300041 Timisoara, Romania; iulia.perva@umft.ro (I.T.P.); adela.chirita@umft.ro (A.C.E.); andreescu.nicoleta@umft.ro (N.A.); maria_puiu@umft.ro (M.P.); mihai.niculescu@gmail.com (M.D.N.); 2 Regional Center of Medical Genetics Timiș, Clinical Emergency Hospital for Children “Louis Țurcanu”, Iosif Nemoianu Street N°2, 300011 Timisoara, Romania; 3 Department of Medical Genetics, Asociatia Oncohelp, 300239 Timișoara, Romania; 4 Department of Gastroenterology and Hepatology, “Victor Babeș” University of Medicine and Pharmacy, Eftimie Murgu Sq., 300041 Timisoara, Romania; renata.fofiu@yahoo.com (R.B.); isporea@umft.ro (I.S.); 5 Center of Advanced Research in Gastroenterology and Hepatology, “Victor Babeș” University of Medicine and Pharmacy, Eftimie Murgu Sq., 300041 Timisoara, Romania; 6 Department of Internal Medicine, “Victor Babeș” University of Medicine and Pharmacy, Eftimie Murgu Sq., 300041 Timisoara, Romania; alexandru.motofelea@umft.ro; 7 Department of Internal Medicine II, Division of Diabetes, Nutrition and Metabolic Diseases, “Victor Babeș” University of Medicine and Pharmacy, Eftimie Murgu Sq., 300041 Timisoara, Romania; sima.alexandra@umft.ro (A.S.); vlad.adrian@umft.ro (A.V.); 8 Center for Research in Preventive Medicine, Faculty of Medicine, “Victor Babeș” University of Medicine and Pharmacy, Eftimie Murgu Sq., 300041 Timisoara, Romania; 9 Center for Molecular Research in Nephrology and Vascular Disease, “Victor Babeș” University of Medicine and Pharmacy, Eftimie Murgu Sq., 300041 Timisoara, Romania; 10 Department of Automation and Applied Informatics, Politehnica University Timișoara, 300223 Timișoara, Romania; cristian.zimbru@upt.ro; 11 Toxicology and Molecular Biology Department, “Pius Brinzeu” Clinical Emergency County Hospital, 300723 Timisoara, Romania; paul.tutac@gmail.com; 12 Advanced Nutrigenomics LLC, Durham, NC 27703, USA

**Keywords:** 5-MTHF, betaine, choline, omega-3 fatty acids, vitamin B12, metabolic syndrome, obesity, FibroScan, controlled attenuation parameter, transient elastography

## Abstract

*Background and Objectives*: The goal of this study was to assess the impact of supplementation with a combination of nutrients on metabolic-dysfunction-associated steatotic liver disease (MASLD)-related liver parameters, and other parameters related to metabolic syndrome in adults with obesity. These measurements included anthropometric and lipid profiling, and FibroScan technology (controlled attenuation parameter (CAP) and transient elastography (TE) values). *Materials and Methods:* A double-blind, placebo-controlled pilot clinical trial was conducted over a three-month treatment period. Adults with metabolic syndrome and obesity were allocated to receive either a cocktail of nutrients with defined daily dosages (5-MTHF, betaine, alpha-linolenic acid, eicosapentaenoic acid, choline bitartrate, docosahexaenoic acid, and vitamin B12) or a placebo. The participants were evaluated at the start and the end of the three-month treatment period. *Results*: A total of 155 participants entered the study, comprising 84 in the treatment group and 71 in the placebo group. The administration of the nutritional supplement resulted in a notable reduction in both CAP and TE scores when compared to the placebo group. The treatment group exhibited a mean reduction in CAP of 4% (*p* < 0.05) and a mean reduction in TE of 7.8% (*p* < 0.05), indicative of a decline in liver fat content and fibrosis. *Conclusions*: The supplementation over a period of three months led to a significant amelioration of liver fibrosis and steatosis parameters in adults with metabolic syndrome and obesity. These findings suggest that this supplementation regimen could be a beneficial adjunct therapy for improving liver health in adults with obesity-induced MASLD.

## 1. Introduction

The prevalence of metabolic syndrome (MetS) is increasing alongside the growing obesity epidemic, resulting in significant healthcare costs. National Health and Nutrition Examination Survey (NHANES) data from 1988 to 2010 indicated that the average BMI and waist circumference (WC) of men and women in the US increased by 0.37% (men) and 0.27% (women) per year, respectively. Thus, in 2016, 11% of men and 15% of women in the total population had obesity [1,2,3,4,5]. 

MetS is a significant concern due to its association with cardiovascular disease (CV) and type 2 diabetes (T2DM), as well as other harmful conditions, such as metabolic-dysfunction-associated steatotic liver disease (MASLD), formerly known as non-alcoholic fatty liver disease (NAFLD), and various types of cancer [4,5,6,7]. There is a strong correlation between MASLD and metabolic syndrome and obesity. Consequently, it represents a significant public health concern due to the potential progression of the disease to more severe liver complications, including fibrosis, cirrhosis, and hepatocellular carcinoma [6,8].

MASLD represents a term that encompasses a wide range of liver conditions that are characterized by the build-up of fat (steatosis) within the liver, which is not a result of excessive alcohol consumption [8]. In the past few decades, the incidence of steatotic liver disease (SLD) and MASLD has increased worldwide, affecting approximately 25–30% of the population [9,10], being the fastest growing cause of liver-related mortality worldwide and becoming an important cause of end-stage liver disease, primary liver cancer, and liver transplantation, which have brought huge health and economic burdens [11].

The pathogenesis of MASLD is multifactorial, involving intricate interactions between genetic predisposition, environmental factors, and metabolic dysregulations. Lifestyle modification, including proper nutrition and regular physical activity, is the therapeutic cornerstone of MASLD treatment [12]. These changes aim to reduce steatosis, chronic inflammation, and fibrosis while managing the primary cardiometabolic risk factors. However, many patients struggle to adopt these long-term changes, highlighting the need for effective pharmacological therapies. Despite numerous studies and clinical trials, no pharmacological treatment has been approved yet worldwide. Some nutraceuticals, such as silymarin [13,14], berberine [15,16], curcumin [17], Nigella sativa [18], Ascophyllum nodosum, Fucus vesiculosus [19], vitamin E, and coenzyme Q10 [20], show promise but require more evidence for long-term efficacy and safety. Additionally, therapies such as incretins and proprotein convertase subtilisin/kexin type 9 (PCSK9) inhibitors, which support treatments for related conditions, such as diabetes and hypertension, have shown potential in reducing the overall cardiometabolic risk [21].

Studies from the last 20 years have underscored the critical role of specific nutrients, such as choline, methyltetrahydrofolate (MTHF), vitamin B12, and omega-3 polyunsaturated fatty acids, specifically eicosapentaenoic acid (EPA) and docosahexaenoic acid (DHA), in modulating key metabolic pathways that influence liver health [2,22,23,24].

The precise and prompt diagnosis of MASLD and the structured management of MASLD patients hold significant importance for the patient’s clinical evolution. Traditionally, liver biopsy has served as the gold standard for identifying histological features indicative of MASLD. However, its invasive nature, limited acceptability, and variability in sampling hinder its widespread use. In recent years, there has been a surge in research focusing on non-invasive diagnostic methods for MASLD, such as the FibroScan-AST score, MRI-AST score, and ADAPT. As a convenient and easily available non-invasive diagnostic method, the controlled attenuation parameter (CAP) has been widely used to evaluate liver fat content [25], being the most validated non-invasive technique for liver steatosis evaluation. A recent meta-analysis conducted by Petroff et al. showed that, among patients with histologically proven MASLD, the area under the curve (AUROC) values of the CAP for assessing the histologically defined steatosis grade were 0.807 for S0 vs. S1–S3, 0.736 (0.720–0.787) for S0–S1 vs. S2–S3, and 0.711 for S0–S2 vs. S3 [26]. Transient elastography (TE) using FibroScan^®^ technology has emerged as a valuable tool in the evaluation of steatotic liver disease due to its non-invasive nature and ability to provide quantitative assessments of liver fibrosis. TE measures liver stiffness, offering clinicians valuable insights into the progression of fibrosis in individuals with steatotic liver disease. This technology enables the detection of early fibrotic changes, allowing for timely intervention and risk stratification [25]. Moreover, fibrosis represents a critical juncture in the advancement of MASLD, and recent studies have underscored the close association between liver fat content and disease progression in MASLD patients [27,28,29].

The present study aims to assess the impact of a micronutrient cocktail supplementation on MASLD in adults with obesity using anthropometric measurements, lipid profiling, and FibroScan technology to measure controlled attenuation parameter and transient elastography values. The working hypothesis in this study was that a defined combination of micronutrients, administered daily, could improve MASLD-related CAP and TE parameters in subjects with obesity.

## 2. Materials and Methods

### 2.1. Study Design

The dataset was obtained from the NutriGen project (using nutrigenomic models to personalize dietary treatments in obesity; ClinicalTrials.gov-NCT02837367) conducted at The Victor Babes University of Medicine and Pharmacy, Timișoara, Romania, between 2016 and 2020. This double-blind, randomized, pilot interventional trial was designed to test the hypothesis that a specific cocktail of nutrients could improve MASLD-related FibroScan parameters in adults with obesity. The NutriGen project also aimed to establish a genetic signature model involved in methyl group donation and omega-6/3 unsaturated fatty acid metabolism, with a high predictive value for the classification of dyslipidemia and insulin resistance in subjects with obesity. The study received ethical approval from The Ethics Committee of The Victor Babes University of Medicine and Pharmacy (No. 6/20 June 2016).

In the initial phase of the project, 400 adults with obesity and dyslipidemia were recruited for a comprehensive biological evaluation, and the methodology of the first phase was previously published [30]. Informed consent was obtained from all adult participants. The study allowed withdrawal of participants at any point, if desired. Anthropometric measurements were registered, and blood samples were taken. The participants were initially composed of 200 adult women and 200 adult men. To be eligible for the study, adults had to meet specific criteria. These included the ages between 18 and 70 years, having a body mass index (BMI) of 30 kg/m^2^ or higher, and an abdominal circumference of 84 cm or more for women and 90 cm or more for men. Additionally, participants had to have confirmed dyslipidemia, which was determined by serum cholesterol levels of 200 mg/dL or higher, HDLc levels of 50 mg/dL or lower for women and 40 mg/dL or lower for men, serum triglycerides of 150 mg/dL or higher, or the use of anti-dyslipidemic treatment, such as statins, fibrates, omega-3 fatty acids, cholestyramine, or ezetimibe. Exclusion criteria for adults were patients with a diagnosis of cancer or medical history of cancer, autoimmune diseases, psychiatric disorders, blood coagulation disorders, and a history of drug or alcohol abuse (assessed using the AUDIT-C questionnaire). For this article, we used the dataset obtained from those participants who underwent the second phase of the project (nutritional intervention). This phase aimed to determine the effectiveness of a dietary supplement combination, based on the findings of the first phase and available medical and scientific data. A total of 240 subjects were recalled from the initial-phase subjects for this part, comprising 120 women and 120 men. The participants were randomized to either a control group receiving placebo, or the treatment group. No further lifestyle modifications were advised, apart from the recommended supplementation. Upon the drop out of some individuals, others were selected from the initial group by randomization. While the target was to obtain a total of 240 compliant participants, the study included 196 participants in its second phase who started the treatment, out of which only 155 participants ended the study and were deemed as compliant. The subjects were invited to undergo anthropometric evaluations, offer a blood sample, and perform a FibroScan evaluation at the beginning of the intervention and after 3 months, at the end of the nutritional supplementation period.

**Figure 1:**
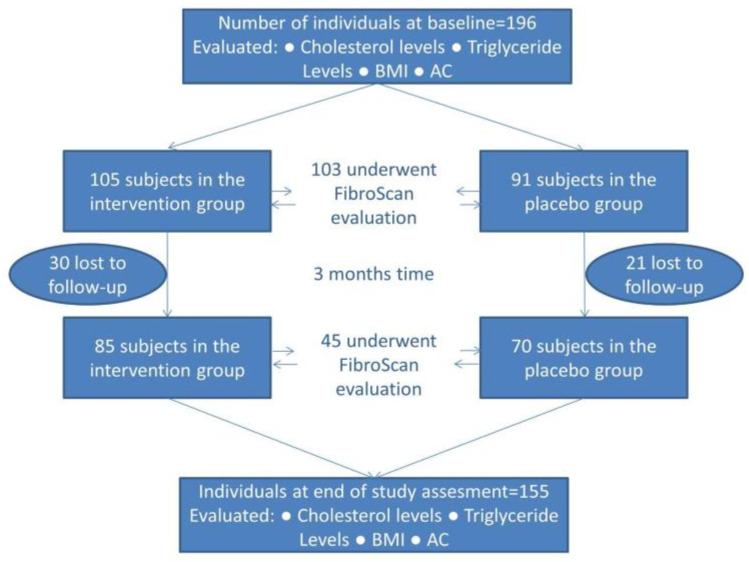
Description of how the study progressed and the parameters evaluated at each stage.

This research was conducted using data pertaining to the adult cohort who were recalled for phase 2 of the study. The dynamic of the sample group is illustrated in Figure 1. A total of 196 patients were included in the baseline evaluation, which entailed the measurement of various physiological parameters, including cholesterol, triglycerides, body mass index (BMI), and abdominal circumference (AC). All patients were recommended a FibroScan evaluation; however, only 103 presented themselves for this procedure. The 196 patients were randomly assigned to either the intervention group (*n* = 105) or the placebo group (*n* = 91). A total of 51 subjects were lost to follow-up, resulting in 155 patients that reached the end of the study (85 in the intervention group and 70 in the placebo). All 155 had undergone anthropometric and serological evaluation at the end point of the study; however, only 45 proceeded to undergo a FibroScan. The number of individuals who underwent both the initial and final FibroScan assessment was 39.

The objective of this study was to evaluate the effectiveness of the intervention related to six parameters: BMI, abdominal circumference (AC), cholesterol levels, triglyceride levels, controlled attenuation parameter, and transient elastography. The pre- and post-intervention values were recorded in standardized units. In some statistical analyses, a percentage score was used. The difference between the baseline and final values was expressed as a percentage change from the baseline value.

### 2.2. Intervention

A total of 196 adults presented for baseline evaluation, anthropometric measurements, and blood sample drawing. They were offered a voucher to use for a FibroScan evaluation and the necessary amounts of supplements for a month of intervention or placebo, depending on the assigned group. The patients were scheduled to attend the supplement administration sessions on a monthly basis. The observation period was three months, during which patients presented themselves monthly to collect the new monthly batches of supplements. Compliance was estimated by counting the number of capsules not used at the end of each month.

The treatment group received the following amounts of micronutrients to be administered daily: 800 mcg of 5-MTHF (5-methyltetrahydrofolate), 2 g of betaine, 1 g of ALA (alpha-linolenic acid), 700 mg of EPA (eicosapentaenoic acid), 500 mg of choline bitartrate, 280 mg of DHA (docosahexaenoic acid), and 1000 mcg of vitamin B12. The participants in the placebo arm of the study were given a placebo that contained low-GI (starch-based) ingredients and corn oil (1 g). The capsules and oils administered to the subjects were identical in appearance, regardless of the group to which they were assigned.

Following the three-month observation period, patients were reassessed. At this juncture, a total of 155 subjects presented for evaluation, and they were all given vouchers for FibroScan evaluation.

### 2.3. Transient Elastography and Controlled Attenuation Parameter

Transient elastography (TE) and controlled attenuation parameter (CAP) evaluations were performed on all participants who used the vouchers, using a FibroScan^®^ Compact 530 device, with either the standard M (3.5 MHz frequency) probe or the XL (2.5 MHz frequency) probe, as appropriate, determined by the automatic probe selection tool. Reliable results were defined as those where the interquartile range interval (IQR) to the median ratio (IQR/M) was less than 30%, based on the median value of 10 valid measurements. Liver stiffness was quantified in kilopascals (kPa), ranging from 2.5 to 75 kPa, while steatosis was quantified in decibels per meter (dB/m), with values ranging between 100 and 400 dB/m [31].

Prior to examinations, participants fasted for at least 4 h. They were positioned supine, with their right arm maximally abducted, following a minimum rest period of 10 min. The probe was positioned between the ribs, aligned parallel to the intercostal space. Subjects were categorized as normal if TE measurements were below 6 kPa and CAP values were below 248 dB/m. Otherwise, if CAP values were above 248 dB/m, they were classified as subjects with steatotic liver disease (SLD). A total of 103 individuals presented themselves at the baseline evaluation, and only 45 did so at the final evaluation, whereas a total of 39 performed both [32,33]. The evaluation was conducted by a number of selected, experienced professionals with expertise in assessing liver parameters.

### 2.4. Cholesterol and Triglycerides

All subjects provided blood samples at baseline and final evaluation, which were collected using tubes with EDTA. The biochemical analysis for determining total cholesterol (CT) and triglycerides (TG) was performed using the Vitros Ortho-clinic 350 equipment (Ortho Clinical Diagnostics Inc., Raritan, NJ, USA), in strict accordance with the manufacturer’s protocols.

### 2.5. Statistical Analysis

Continuous variables were tested for normal distribution. Means were presented as plus/minus standard error (SE). Results for statistical testing using non-normally distributed data were presented as median with interquartile for 25% and 75% of measurements (Q1 and Q3), respectively. The normality of the distribution was evaluated using the Shapiro–Wilk test. Differences between groups for normally distributed continuous data were assessed using Welch’s *t*-test for two groups or ANOVA for more than two groups. Post hoc analyses, when necessary, were performed using the Bonferroni correction to adjust for multiple comparisons. For non-normally distributed continuous data, the Mann–Whitney U test and the Wilcoxon signed-rank test were used for two-group comparisons, while the Kruskal–Wallis test was applied for comparisons involving three or more groups. The false discovery rate (q-value) was applied to adjust for multiple comparisons in the Mann–Whitney U test, Wilcoxon signed-rank test, and Kruskal–Wallis test. Categorical data were reported as frequencies (n) and percentages (%). A prior power analysis was performed with at least 80% statistical power with the 95% confidence interval. All statistical analyses were performed using R Studio version 3.6.0 using the packages stats, dplyr, coin, multcomp, and pwr.

A detailed account of the baseline characteristics of the individuals who participated in the research study can be found in Table 1. This table provides a succinct overview of the principal parameters, including clinical and biochemical data, which were collected at the outset of the study. In order to provide a comprehensive profile of the cohort, a range of parameters were selected for analysis, including BMI, AC, cholesterol, triglycerides, TE, and CAP values. The comprehensive baseline data were essential for understanding the original state of the study population, conducting subsequent analyses of intervention effects, and ensuring that any observed outcomes could be accurately attributed to the interventions and not to pre-existing disparities among participants. By presenting the aforementioned baseline characteristics, Table 1 serves as a foundational reference point, ensuring that the study’s results are comparable and reliable.

## 3. Results

Participants were divided into two groups: those receiving a nutritional intervention (treatment group, *n* = 84, 54%) and those receiving a placebo (placebo group, *n* = 71, 46%). The median age of participants was 58 years, with an interquartile range (IQR) of 49 to 63 years. The cohort comprised 69 females (45%) and 86 males (55%). Notable findings included an age difference between genders, with a median age of 55 years for males and 59 years for females, but this difference was not found to be statistically significant (*p* = −0.262).

The Wilcoxon signed-rank test results in the placebo group indicated a statistically significant increase in the AC measurement, with a *p*-value of 0.003. Other measurements, including cholesterol, triglycerides, BMI, CAP, and TE, did not exhibit significant changes before and after the placebo intervention (Table 2). Data were calculated as the difference between measured values at the end and start of the intervention in a paired analysis. 

For the treatment group, the Wilcoxon signed-rank test results indicated statistically significant decreases in both BMI and CAP measured values following the intervention, with *p*-values of <0.001 and 0.037, respectively (*p* < 0.05). No significant changes were observed in cholesterol, abdominal circumference, triglycerides, or TE values (Table 3).

**Figure 2:**
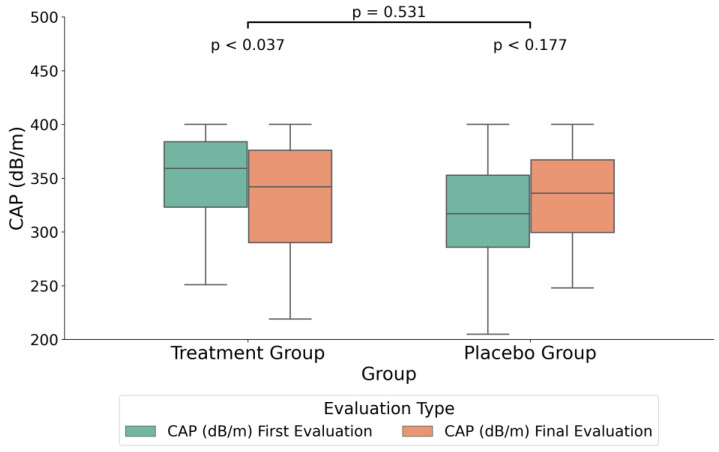
Boxplot illustrating the median differences in controlled attenuation parameter (CAP) measurements (dB/m) between the treatment and placebo groups. The plot includes the CAP values at both the initial measurement and the final treatment measurement. The boxplot visualizes the interquartile range (IQR) with the median, and the whiskers indicate the minimum and maximum values within 1.5 times the IQR from the lower and upper quartiles. Outliers are represented as individual points beyond the whiskers.

**Figure 3:**
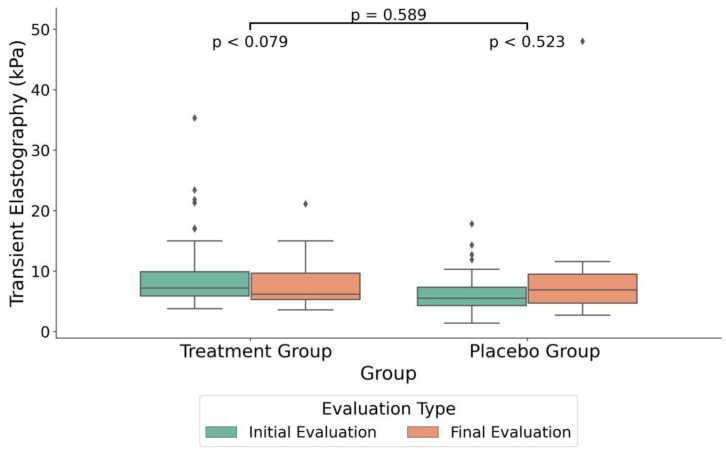
Boxplot illustrating the median differences in transient elastography (kPa) between the treatment and placebo groups. The plot includes the TE values at both the initial measurement and the final treatment measurement. The boxplot visualizes the interquartile range (IQR) with the median, and the whiskers indicate the minimum and maximum values within 1.5 times the IQR from the lower and upper quartiles. Outliers are represented as individual points beyond the whiskers.

In order to assess potential differences between the treatment and placebo groups (Table 4), all data were expressed as percent change between the end and start of the study in order to take into account the individual variability of these parameters. Therefore, the variations between the initial and final measurements, expressed in standardized units, were calculated as the percentages of change from the start values. The intervention group had a significantly greater median decrease in the CAP compared to the placebo group, with medians of −4.0% and 5.4%, respectively (F (1,37) = 7.14, *p* = 0.013, FDR = 0.05, see also absolute values in Figure 2). Additionally, age distribution varied significantly between the groups (F (1,152) = 24.12, *p* < 0.013, FDR < 0.001). Furthermore, TE showed a significant median decrease of –7.8% in the treatment group when compared to a median increase of 8.6% in the placebo group (F (1,37) = 4.49, *p* = 0.043, FDR = 0.11, see also absolute values in Figure 3).

In the treatment group, the initial median measurement of CAP was 355 dB/m (SD = 35.57) and the final median measurement was 332 dB/m (SD = 51.76), with a *p*-value of 0.037. In the placebo group, the initial measurement was 318 dB/m (SD = 53.51), versus 334 dB/m (SD = 47) in the final evaluation, without a significant statistical difference, as indicated by a *p*-value of 0.177 (Figure 2).

In the treatment group, the initial TE measurement was 9.08 kPa (SD = 4.63) and the final measurement was 8.02 kPa (SD = 4.47), but the change was not statistically significant (*p* = 0.079). In the placebo group, the initial measurement was 6.36 kPa (SD = 3.46), versus 6.69 kPa (SD = 2.67) in the final evaluation, without a significant statistical difference, as indicated by a *p*-value of 0.523 (Figure 3). However, when comparing the percent change for both CAP and TE between the two groups, the percent changes were significant for both measurements (Table 4).

We conducted a gender analysis within the treatment group and found significant differences in BMI changes over time for both females and males. For females, the mean BMI decreased from 39.03 kg/m^2^ (SE = 1.432) to 37.80 kg/m^2^ (SE = 1.428), indicating a significant mean change of 0.951 kg/m^2^ (SE = 0.466, *p* = 0.007). Similarly, for males, the mean BMI decreased from 34.03 kg/m^2^ (SE = 1.00) to 33.44 kg/m^2^ (SE = 1.03), with a significant mean change of 0.511 kg/m² (SE = 0.264, *p* = 0.048). A significant difference was found in the decrease in the mean value in the gender analysis in the treatment group for the transient elastography measurements. For female participants, the mean decreased from a baseline of 7.47 kPa (SD = 3.84) to a final measurement of 5.83 kPa (SD = 2.05), with a significant difference (*p* = 0.042). In the male analysis, the mean decreased from a baseline of 10.28 (SD = 4.95) to a final measurement of 9.66 (SD = 5.13), but with no statistical significance (*p* = 0.505).

## 4. Discussions

This study indicated that the daily administration of a micronutrient cocktail for three months, in adults with obesity, improved both the CAP and TE parameters, as measured using the FibroScan method. While in the placebo group, both parameters did not register statistically significant changes (Table 2), in the treatment group, the CAP decreased by 19 dB/m, and this change was statistically significant (Table 3). When both parameters were quantified as percent change from baseline (start of the study, Table 4), the statistical comparison between groups indicated that both the CAP and TE registered statistically significant reductions in the treatment group, by median values of 5.4% and 8.6%, respectively. These significant differences between groups suggested that the administration of a specific mix of micronutrients (see the Materials and Methods Section) may have a positive impact on the reduction in MASDL in adults with obesity, as well as on the possible reduction in BMI.

It is already known that a series of interventions, such as therapeutic interventions or lifestyle changes, can lead to the improvement of CAP values and liver fibrosis values evaluated with TE. Studies have indicated a decrease in liver stiffness values measured with TE after the administration of etiological antiviral treatment in subjects with chronic HBV or HCV hepatopathy [34,35,36,37], as well as a reduction in TE values in subjects with chronic alcohol-induced liver disease in subjects who have withdrawn from alcohol consumption [38,39,40].

Regarding the patients with MASLD, studies have shown that certain diets, such as the Mediterranean diet [41] or the Eight-Week Very-Low-Calorie Ketogenic Diet (VLCKD) [42], led to a reduction in hepatic steatosis quantified by CAP, as well as to a reduction in liver stiffness values evaluated by TE. In addition to diet and physical activity, a series of other interventions, such as therapeutic interventions, have been studied to reduce CAP and TE values. A recent study [43] showed that the administration of sodium-glucose cotransporter-2 inhibitors improved hepatic fibrosis by ameliorating hepatic steatosis and inflammation. Another study, published in 2024 [44], evaluated the benefit of silymarin versus essential phospholipids in reducing CAP and TE values in subjects with MASLD, and it showed that both silymarin and essential phospholipids induced a reduction in CAP values, while only essential phospholipids had effects on reducing liver stiffness values.

This study is the first, to our knowledge, to indicate that a cocktail of micronutrients consisting of 5-methyltetrahydrofolate, betaine, alpha-linolenic acid, eicosapentaenoic acid, choline, docosahexaenoic acid, and vitamin B12 may be beneficial toward the reduction of MASLD-specific FibroScan parameters. If further validated in larger studies, these findings would allow for effective nutritional solutions aimed at the reduction of obesity-induced MASLD. Since none of the participants were guided for other nutritional interventions, it is plausible to consider that the cause of these reductions was the administration of the aforementioned micronutrients. The daily dosages used were carefully considered after a thorough review of the relevant literature on the roles of these micronutrients in the possible reduction of MASLD.

The micronutrients selected to be administered are already known to play different mechanistic roles in the attenuation of MASLD. A growing body of evidence supports that supplementation with omega-3 or methyl donors decreased steatosis, but a correlation with decreasing liver fibrosis was challenging to prove, as indicated by several studies [45,46,47,48,49,50].

Previous studies have indicated that methyl donors play a pivotal role in maintaining liver health. Deficiencies in these nutrients have been associated with the development of fatty liver and liver damage [51,52,53]. 

In the results of the initial phase of our study, which focused on the assessment of dietary intakes, we reported that our participants had suboptimal intakes of choline and folates [53]. It is known that choline deficiency impacts the synthesis of very-low-density lipoprotein (VLDL), which is crucial for triglyceride secretion from the liver. It has been hypothesized that the hepatic fat accumulation observed in MASLD could be, in part, the result of impaired lipid clearance, rather than impaired de novo lipogenesis [54]. It can thus be speculated that within our cohort of participants with low choline intakes, the supplementation provided the intake to optimal levels, allowing the liver to clear lipids into VLDL, which in turn resulted in a reduction in fat deposits and a subsequent reduction in steatosis. It is noteworthy that in our cohort, although the supplementation may be linked to a reduction in liver steatosis, changes in serum triglycerides did not occur, as previously suggested [51,55,56]. Chai et al. demonstrated an inverse correlation between optimal dietary choline intake and the CAP score. Consequently, an association was established between low CAP values in individuals with MASLD and high choline intake [2]. 

One interesting observation was the median age of females within our cohort. A median age of 59 could suggest that most females recruited were in the menopausal phase of life. It can thus be speculated that the endogenous biosynthesis of choline (via the diethanolamine pathway), an estrogen-dependent process previously demonstrated in fertile women, was poorly represented in our cohort [57,58]. This could explain the greater response observed in women’s TE measurements, as their choline-deficient diets may affect them later in life. Therefore, it can be speculated that the liver fibrosis could be less advanced in females, thus facilitating better repair than in males. 

Because betaine is the oxidation product of choline, which then donates a methyl group for methionine synthesis (used for the endogenous synthesis of choline), it may be reasonably speculated that the addition of betaine to the mix is based on plausible mechanisms that could contribute to a MASLD reduction [52,53,59].

We have previously indicated that the intakes of folates were inadequate in our cohort [53]. The impact of 5-methyltetrahydrofolate (5-MTHF) deficiency or folates on the hepatic tissue has been previously linked to the impairment of one-carbon transfer reactions in rodents and with the emergence of a MASDL phenotype. Consequently, 5-MTHF has been proposed as a potential therapeutic avenue for the alleviation of liver steatosis [60,61]. Because 5-MTHF also contributes to the methylation of homocysteine to methionine (along with vitamin B12 as a cofactor), the addition of this micronutrient was justified, as it also contributes to the methylation pathways, similar to the role of betaine [62].

As early as 2005, Alwayn et al. demonstrated that n-3 PUFA supplementation could protect against hepatic steatosis in a murine model of parenteral nutrition, where all animals typically develop steatosis and liver enzyme disturbances [63]. In 2008, Xin et al. suggested this association as a therapy for non-alcoholic fatty liver disease [64]. Sokal-Dembowska et al. [46] reviewed the implications of the selected nutraceuticals, including vitamin C, beta-carotene, omega-3 fatty acids, and curcumin. They indicated that incorporating these ingredients into the treatment of patients with MASLD/MASH, alongside behavioral and pharmacological therapy, may help combat inflammation, reduce oxidative stress, and prevent liver damage. In their Annual Review of Nutrition [65], Spooner and Jump suggested that MASLD may be considered a disease of ω3 PUFA deficiency, particularly at the hepatic level [65]. This deficiency specifically relates to docosahexaenoic acid (DHA) and eicosapentaenoic acid (EPA), owing to their antioxidant and anti-inflammatory properties [66]. As anticipated and in line with these findings, our cohort did not meet the recommended dietary allowances (RDA) for EPA and DHA according to the United States Department of Agriculture (USDA) [67] and European Food Safety Authority (EFSA) [68] guidelines, with participants showing inadequate intakes [53]. This evidence supports a further connection with our MASLD results, which demonstrated a significant decrease in CAP and TE within the nutritional group, and it suggests that the addition of EPA and DHA may have been beneficial. ALA is a plant-based omega-3 fatty acid precursor essential for human health, and it is obtained exclusively through diet, as the body is not able to synthesize it. Previously, the anti-inflammatory effects of omega-3 fatty acids were attributed solely to EPA and DHA, with ALA seen primarily as a precursor or energy source [69]. However, recent research highlighted ALA’s health benefits, particularly for metabolic health and the management of MASLD [70]. ALA directly modulated the immune response pathways by activating GPCRs, such as GPR120, reducing the pro-inflammatory cytokine production and attenuating the inflammation in various cell types [71,72]. Thus, ALA is considered to prevent the progression of simple steatosis to steatohepatitis [73].

In terms of metabolic benefits, n-3 PUFAs help lower plasma triglyceride levels, particularly in cases of hypertriglyceridemia, by inhibiting the synthesis of total cholesterol, triglycerides, and very-low-density lipoprotein in the liver. A meta-analysis performed by Lee et al. in 2020 on the effects of n-3 PUFA supplementation in MASLD offered interesting data. Their findings pointed toward a significantly diminished liver fat accumulation, as compared with the placebo, and to improvement in the levels of triglycerides, total cholesterol, and BMI [74], characteristics shared by our cohort only with respect to steatosis and fibrosis (CAP and TE; Table 4). This could be attributed to varying assessment methods and study durations in the meta-analysis. Most studies combined interventions with lifestyle changes and investigated over at least six months. Based on these observations, we assume that an extended period of more than three months would have led to improvements in these parameters. An intriguing hypothesis for future research is to stratify MASLD into different stages of progression when evaluating the benefits of supplementation with each of the three main n-3 PUFAs. Yang et al. suggest that n-3 PUFA supplementation may be effective in the early stages of MASLD, but not in patients with more severe MASLD or MASH [75]. On the other hand, a recent study on the mechanisms of liver fibrosis and the role of nutraceuticals in treating MASLD/MASH [45] highlighted and supported previous evidence of their significant role in reducing liver fibrosis by promoting liver regeneration [76,77].

### Limitations

While the study was initiated with an expected cohort size of over *n* = 100 per group, reduced compliance and voluntary withdrawal drastically reduced the number of participants that underwent FibroScan measurements. Therefore, this study can be considered as a pilot, and future studies should better and more comprehensively address the hypothesis that the administration of micronutrients may be beneficial to reducing MASDL. Because of the reduced follow-up, randomization for all six parameters was not possible (Table 1). Therefore, the statistical significance of the results reported in this study should be considered with caution. In order to minimize the risk of reporting spurious results, FDRs were also assessed for our final comparisons. The small sample size did not allow a comprehensive stratification for sex, age, and other variables.

The study included participants aged 18–70, which represents a broad age range that warrants consideration of the distinctive physiological and metabolic characteristics associated with various stages of adulthood and old age. It is essential to acknowledge that such an expansive age span has the potential to introduce bias due to age-related factors that may influence the outcomes. However, considering the limited sample size, data stratification regarding age-specific variations yielded inconclusive results.

## 5. Conclusions

The administration of a combination of methyl donors (choline, betaine, and 5-MTHF), omega-3 fatty acids (EPA, DHA, and ALA), and vitamin B12 over a period of three months resulted in a statistically significant improvement in liver fibrosis and steatosis parameters in adults with metabolic syndrome and obesity. The results suggested that supplementation with a defined combination of micronutrients could be causally related to the improvements observed for CAP and TE parameters. These findings suggest that this combination may be a valuable complementary treatment for improving liver health in the obese adult population. However, further investigation is required to confirm these findings in a larger cohort.

## Figures and Tables

**Table 1:** Baseline characteristics in the treatment and placebo groups (prior to treatment start).

Characteristic	N	Treatment N = 84 (54%)	Placebo N = 71 (46%)	*p*-Value	q-Value
Cholesterol (mg/dL)	155	193 (154, 216)	188 (160, 233)	0.58	0.60
Triglycerides (mg/dL)	155	168 (126, 232)	177 (140, 224)	0.60	0.60
Body mass index (kg/m^2^)	154	35.0 (31.4, 40.6)	33.7 (30.7, 36.9)	0.084	0.17
Abdominal circumference (cm)	154	118 (111, 126)	118 (108, 126)	0.44	0.60
CAP (dB/m)	103	359 (323, 384)	317 (286, 353)	0.004	0.011
Transient elastography (kPa)	103	7.2 (5.9, 9.9)	5.5 (4.3, 7.3)	<0.001	0.002

Median values (Q1, Q3) are presented for each group. *p*-values were obtained using the Wilcoxon rank sum test, with q-values indicating the false discovery rate for multiple testing. CAP—controlled attenuation parameter, and N represents the number of participants (total and in each group).

**Table 2:** Wilcoxon signed-rank test for before and after measurements in the placebo group.

Characteristic	N ^1^	Initial Value ^2^	Final Value	*p*-Value
Cholesterol (mg/dL) ^3^	51	188 (160, 233)	222 (176,256)	0.167
AC (cm) ^3^	46	118 (108, 126)	113 (108, 122)	0.003
Triglycerides (mg/dL) ^3^	51	177 (140, 224)	179 (120, 264)	0.729
BMI (kg/m^2^) ^3^	45	33.7 (30.7, 36.9)	32 (30.7, 36.7)	0.121
CAP (dB/m) ^3^	18	317 (286, 353)	336 (300, 367)	0.177
TE (kPa) ^3^	18	5.5 (4.3, 7.3)	6.9 (4.7, 9.5)	0.523

^1^ Number of measurements (end vs. start of intervention). ^2^ Median values (Q1, Q3) are presented for each group. ^3^ Change from baseline. AC—abdominal circumference, BMI—body mass index, CAP—controlled attenuation parameter, and TE—transient elastography.

**Table 3:** Wilcoxon signed-rank test for before and after measurements in the treatment group.

Characteristic	N ^1^	Initial Value ^2^	Final Value	*p*-Value
Cholesterol (mg/dL) ^3^	59	193 (154, 216)	190 (154, 234)	0.803
AC (cm) ^3^	58	118 (111, 126)	118 (109 126)	0.081
Triglycerides (mg/dL) ^3^	59	168 (126, 232)	177 (119, 235)	0.966
BMI (kg/m^2^) ^3^	58	35.0 (31.4, 40.6)	34.0 (30.6, 39.8)	<0.001
CAP (dB/m) ^3^	21	359 (323, 384)	342 (290, 376)	0.037
TE (kPa) ^3^	21	7.2 (5.9, 9.9)	6.2 (5.3, 9.7)	0.079

^1^ Number of measurements (end vs. start of intervention). ^2^ Median values (Q1, Q3) are presented for each group. ^3^ Change from baseline. AC—abdominal circumference, BMI—body mass index, CAP—controlled attenuation parameter, and TE—transient elastography.

**Table 4:** Changes in the parameters between the treatment and placebo groups at the end of the intervention.

	N ^2^	Treatment ^3^	Placebo ^3^	WSRT ^4^	FDR ^5^
Cholesterol ^1^ (%)	110	−22.5 −3.6 31.6	−7.3 2.0 21.6	*p* = 0.31	0.41
Triglycerides ^1^ (%)	110	−32.9 −1.2 52.9	−31.9 3.0 43.1	*p* = 0.91	0.91
AC ^1^ (%)	104	−2.7 0.0 0.9	−4.0 −1.8 0.9	*p* = 0.15	0.34
BMI ^1^ (%)	103	−4.4 −2.0 1.2	−3.4 −1.3 1.1	*p* = 0.21	0.3
CAP ^1^ (%)	39	−10.0 −4.0 1.4	−4.6 5.4 15.5	*p* = 0.01	0.05
TE ^1^ (%)	39	−33.4 −7.8 1.7	−14.8 8.6 30.5	*p* = 0.04	0.11

Detailed analysis of the progression of cholesterol, triglycerides, abdominal circumference (AC), body mass index (BMI), controlled attenuation parameter (CAP), and transient elastography (TE) throughout the three-month nutritional supplementation. ^1^ Numbers represent percentage change at the end of the study, from the start value. ^2^ Number of subjects that underwent both initial and final evaluation in regard to the parameter. ^3^ Percent change flanked by Q1 and Q3 parental values. ^4^ WSRT—Wilcoxon signed-rank test. ^5^ False discovery rate correction for multiple testing for six variables.

## Data Availability

The dataset is available upon request from the authors. The raw data supporting the conclusions of this article will be made available by the authors upon request.
